# Role of oxidative stress in pathogenesis of metabolic syndrome 

**Published:** 2012

**Authors:** Soleiman Mahjoub, Jila Masrour-Roudsari

**Affiliations:** 1 Fatemeh Zahra Infertility and Reproductive Health Research Center, Babol University of Medical Sciences, Babol, Iran.; 2 Department of Biochemistry, Faculty of Medicine, Babol University of Medical Sciences, Babol, Iran.; 3 nfectious Diseases and Tropical Medicine Research Center, Babol University of Medical Sciences, Babol, Iran.

**Keywords:** Oxidative stress, Metabolic syndrome, Pathogenesis, Diabetes, Cardiovascular diseases.

## Abstract

The metabolic syndrome (MS) recognized as a major cause of type 2 diabetes and cardiovascular diseases, has become one of the major public health challenges worldwide. The pathogenesis of the metabolic syndrome is multiple and still poorly understood. No single factor has yet been identified as an underlying causal factor. There is a growing belief, however, that obesity, especially visceral obesity, may play an important role in the development of the syndrome. Visceral adiposity seems to be an independent predictor of insulin sensitivity, impaired glucose tolerance, dyslipidemia and elevated blood pressure. An increasing number of studies confirm that oxidative stress, chronic inflammation and angiogenesis all play important roles in the pathogenesis of MS. Chronic hyperglycemia causes oxidative stress in tissues prone to complications in patients with diabetes. Oxidative stress occurs in a cellular system when the production of free radical moieties exceeds the antioxidant capacity of that system. If cellular antioxidants do not remove free radicals, radicals attack and damage proteins, lipids, and nucleic acids. The oxidized or nitrosylated products of free radical attack have decreased biological activity, leading to loss of energy metabolism, cell signaling, transport, and other major functions. These altered products are also targeted for proteosome degradation, further decreasing cellular function. Accumulation of such injury ultimately leads a cell to die through necrotic or apoptotic mechanisms. In conclusion, a puzzle of many pieces of evidence suggests that free radical overgeneration may be considered the key in the generation of insulin resistance, diabetes, and cardiovascular disease.

Oxygen is vital for most organisms, but, paradoxically might be the source of molecules to damage key biological sites. Reactive oxygen and nitrogen species (RONS) are continuously produced in the body as by-products of the reaction leading to energy production through the mitochondrial and microsomal electron-transport chains. Other endogenous sources of RONS* in vivo *are oxidative bursts in phagocytes and enzyme systems such as xanthine oxidase and cytochrome P-450 oxidase. Exogenous sources are represented by cigarette smoking, pollution, physical exercise etc. Physiological levels of RONS are crucial for a proper cell function (i.e. intracellular signaling, inflammation and immune function); problems arise when RONS levels largely exceed their physiological concentration leading to oxidative stress. Oxidative stress, the shift of the redox balance through oxidative potentials, may damage biological molecules altering cell function and leading to cell death. Antioxidants, molecules that inhibit or prevent oxidation of a substrate, evolved to protect biological systems against damage induced by RONS. An increasing number of studies confirm that oxidative stress play important roles in the pathogenesis of different diseases. 

The mechanisms of oxidative stress in animal models and status of oxidative stress in different diseases were investigated by Mahjoub et al. in the population of Babol, North of Iran (1-12). Also, the prevalence and risk factors of metabolic syndrome in the population of Babol were investigated by Mahjoub and colleagues (13, 14). 

The metabolic syndrome (MS), a cluster of several risk factors for diabetes and cardiovascular disease, major causes of morbidity and death, is a highly prevalent condition in the world. Ethiopathogenesis of MS is highly complex and participating factors multiple and highly varied. Despite the availability of diverse treatment tools and methodologies, the problem persists in its increasing tendency. This fact shows the necessity to improve the knowledge on MS, both in its overall complexity and in its biochemical mechanisms. An increasing number of studies confirm that oxidative stress, chronic inflammation and angiogenesis all play important roles in the pathogenesis of the MS. Although many growth factors and cytokines have been reported to interfere somehow in those entities, the precise interplay of those effectors among them and towards the MS is not yet clear. Most of the published studies focus on the epidemiology, clinical symptoms, association between features of MS, or the respective prevention/treatment strategies (14-17).

In the present review, the knowledge gathered on MS as a whole, as well as on the implication of mechanisms of oxidative stress, chronic inflammation and angiogenesis in its development and progression is critically reviewed and discussed. It, thus, allows an integrated view of the condition, favoring a holistic approach towards preventive and therapeutic possibilities. 

Chronic hyperglycemia causes oxidative stress in tissues prone to complications in patients with diabetes (18, 19). Diabetes is an epidemic in developed countries. In the United States, 16 million individuals are diabetic, and the number is increasing at a rate of 5% per year. The major form of diabetes in the population is type 2, which accounts for up to 95% of diabetes cases in the United States (20). Among children, type 1 diabetes poses a greater risk, although this may change in the future because the rate of type 2 diabetes in children and adolescents is increasing (21). The microvascular complications of diabetes carry a high morbidity and, when coupled with macrovascular complications, high mortality results (22).

Several free radical species are normally produced in the body to perform specific functions. Superoxide (O2^_.^), hydrogen peroxide (H2O2), and nitric oxide (NO) are three free radical reactive oxygen species (ROS) that are essential for normal physiology, but are also believed to accelerate the process of aging and to mediate cellular degeneration in disease states. These agents together produce highly active singlet oxygen, hydroxyl radicals, and peroxynitrite that can attack proteins, lipids, and DNA. 

***A. Superoxide O2*** ^_.^

Superoxide O2^_.^is generated by the mitochondrial electron transfer chain during the oxidation of reduced nicotinamide adenine dinucleotide (NADH) to oxidized nicotinamide adenine dinucleotide (NAD)^+^ and also as a by-product of many enzymes that act as oxidases. Approximately 4% of electrons that enter the respiratory chain lead to the formation of O2^_.^ (23). The beneficial effects of O2^_.^include regulation of vascular function, cell division (24), inflammation (25), apoptosis (26), and bactericidal activity of neutrophils (27). Decreased levels of O2^_.^can lead to an increased susceptibility to bacterial infections, as illustrated in Down’s syndrome patients with elevated cytoplasmic superoxide dismutase (SOD)1 (28). Thus, cellular levels of O2^_.^are under tight regulation. Excess O2^_.^is removed through the activity of a family of SOD enzymes that convert O2^_.^to H2O2 and oxygen.O2^_.^ overproduction occurs in complication-prone tissues when cellular metabolism is perturbed by excess glucose.

ATP synthase is inhibited, and electron transfer slows. This can cause overproduction of O2^_.^ in two ways. First, the half-life of highly reactive quinone intermediates is prolonged, increasing the release of electrons to combine with molecular oxygen and form O2^_.^. Second, when electron transfer no longer can regenerate NAD^+^, the enzyme NADH oxidase is activated and generates O2^_. ^as a byproduct.

***B. Hydrogen peroxide (H2O2)***

H2O2 is produced after the spontaneous or SOD-catalyzed dismutation of O2^_.^ as well as many other enzymatic reactions. Unlike O2^_.^, which remains at the site of production, H2O2 can diffuse across membranes and through the cytosol (29). This ROS is another component of leukocyte-mediated defense against bacteria. Because H2O2 is a powerful oxidizing agent, cells express abundant catalase, glutathione (GSH), and thioredoxin (Trx) that convert H2O2 to water. When H2O2 reacts with free Fe2^+^, the iron is oxidized and hydroxyl radicals are produced. There are many severe consequences of hydroxyl radical production, including loss of vasodilation that can lead to endothelial injury and tissue hypoxia (30). 

Hyperglycemia activates many signaling mechanisms in cells. Four major pathways that can lead to cell injury downstream of hyperglycemia are illustrated. 1) Excess glucose shunts to the polyol pathway that depletes cytosolic NADPH and subsequently GSH. 2) Excess glucose also undergoes auto oxidation to produce AGEs that impair protein function and also activate RAGEs that use ROS as second messengers. 3) PKC activation both further increases hyperglycemia and also exacerbates tissue hypoxia. 4) Overload and slowing of the electron transfer chain leads to escape of reactive intermediates to produce O2^_. ^as well as activation of NADH oxidase that also produces O2. A unifying mechanism of injury in each case is the production of ROS that impair protein and gene function. TCA, Trichloroacetic acid; PAI-1, plasminogen activator inhibitor-1 (figure 1) (31).

**Figure 1:**
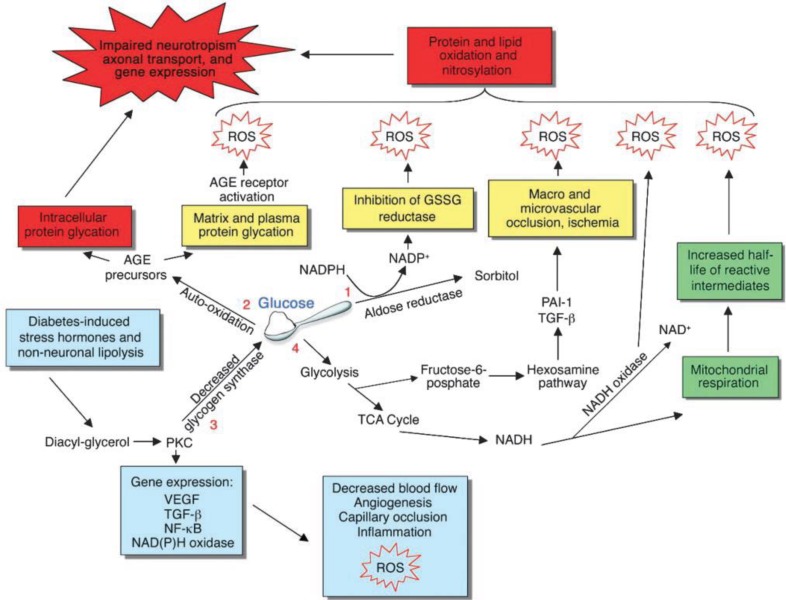
Four major pathways that hyperglycemia can lead to cell injury via oxidative stress (31).

***C. Nitric oxide (NO)***

NO is generated through the activity of a cytosolic enzyme known as NO synthase (NOS). There are both constitutively expressed calcium-dependent isoforms of NOS and an inducible isoform that is associated with inflammation and cell activation (32). NO plays a major role in regulating vascular tone by activating soluble guanylate cyclases that regulate ion channels. In addition, NO modulates cellular respiration through direct inhibition of cytochrome oxidase by competitively occupying the oxygen-binding site (33). The inducible form of NOS is increased in the arteries of diabetic rats (34). Damaged neurons recover more slowly in the presence of NO, and conversely, NOS inhibitors promote neuronal recovery from injury (35). NO is also believed to act as a neurotransmitter (36). The dual role of NO as both beneficial and detrimental is illustrated in stroke models. Under ischemic insult, endothelial NO produces vasodilation that can improve blood flow, but neuronal NO is produced down stream of calcium dysregulation and can prevent energy generation in the mitochondria (37). More importantly, NO acts as an antioxidant in certain environments and prevents lipid peroxidation (38). However, when O2^_. ^increases, NO reacts with the O2^_. ^to form peroxynitrite and becomes a prooxidant.

***Cellular Injury through Excess ROS Production***

The production of ROS is under tight control in healthy cells, but overproduction during metabolic dysfunction leads to cellular injury. Although both O2^_.^ and NO are relatively inert, when they combine they form the highly reactive peroxynitrite that attacks and inhibits proteins and lipids. In addition, both O2^_.^ and NO can attack iron-sulfur centers of enzymes and other proteins to release iron atoms and consequently inhibit enzyme/protein activities. There are many important proteins that are exquisitely sensitive to this type of inhibition including complexes I–III of the electron transfer chain, aconitase of the trichloroacetic acid cycle, and biotin synthase (39, 40). The formation of lipid, protein, and nucleic acid adducts involves a complex chain reaction using a range of biological substrates that contain reactive methylene groups. Intermediates in the chain reaction can have extremely high oxidative ability and so cellular damage can be extensive. The chemistry of these reactions has been reviewed previously (41, 42). Lipids present in plasma, mitochondrial, and endoplasmic reticulum membranes are major targets of ROS attack and peroxidation. End products of lipid peroxidation, known as lipid peroxides, can be toxic to a cell and require removal by GSH as described below. Similarly, proteins and nucleic acids can be subject to peroxidation and nitrosylation. Although these end products are not usually directly toxic to the cell, accumulation of inactive proteins can overload the ability of a cell to recycle them, and damage of DNA is known to activate the mechanisms of apoptosis. In addition, accumulation of modified proteins decreases their function, leading to severe loss of normal activity. Axonal transport can be slowed, leading to decreased delivery of growth factors and intermediates from the synapse to the cell body and resulting in induction of apoptosis (43). Oxidative modification of transcription factors not only leads to decreased expression of many proteins such as apoptosis inhibitory factor, complex I, and Bcl-2, but also results in increased expression of stress proteins that may be proapoptotic, including cyclooxygenase 2, poly-ADP ribose polymerase, and Jun kinase (JNK) (44–47). Production of ROS in all cells not only results in deleterious events but also can play a role in differentiation and development. Redox status can have profound effects on gene expression, so that oxidative stress increases growth factors, stress response elements, and apoptosis pathways (48). In contrast, certain proteins including cytokines, cytochrome c oxidase, and enzymes involved in glucose respiration are repressed by oxidative stress signaling (49). Understanding of gene regulation by reactive oxygen intermediates is rapidly expanding. Once the mechanisms are more fully understood, the ability of a cell to respond to stress by changing gene expression may provide an important therapeutic target. The most significant consequence of oxidative stress in dividing cells may be DNA modifications that produce genomic instability and mutations (50). Nondividing neurons may suffer less from oxidative damage of DNA. Yet, mitochondrial DNA is particularly sensitive to oxidative damage (51), which would impair energy regulation and thus would be critically important in high energy-requiring neurons. Oxidative stress-mediated neuronal degeneration is implicated in several types of neurodegenerative disease (52–54). In nondividing cells like neurons, damage to proteins and lipids may be more injurious than DNA damage, because this may render proteins unable to perform axonal transport and signaling (43). For example, synaptosomal membranes as well as cytosolic proteins become oxidized, and these changes can be correlated to alterations in brain function (55). Loss of function in neurons rapidly promotes necrotic or apoptotic mechanisms (53, 56).

In the past few decades, type 2 diabetes mellitus (T2DM) has rapidly increased in the world. It has been estimated that the number of diabetic patients will become more than double within 15 years (57). Moreover, although T2DM was previously considered a slow-onset disease of middle-aged and older subjects, an emerging issue is the recent increase in diagnoses of T2DM and prediabetic conditions in children (58). T2DM is mainly characterized by the development of increased morbidity and mortality for cardiovascular disease (CVD) (59), so that it is suggested that diabetes may be considered a cardiovascular disease (60). However, CVD risk is elevated long before the development of diabetes (61). The close relationship between T2DM and CVD has led to the “common soil” hypothesis (62), postulating that T2DM and CVD share common genetic and environmental antecedents. One of the most important roles of these possible antecedents is considered insulin resistance. In genetically predisposed subjects, the combination of excess caloric intake and relatively scarce physical activity, with the likely consequence of obesity, can induce a state of resistance to the action of insulin (63). Insulin resistance is an important component of the metabolic syndrome, first described as a clinical syndrome in which the clustering of factors such as obesity, dyslipidemia, and hypertension leads to a substantial increase in CVD risk (64). Insulin resistance is also a crucially important metabolic abnormality in T2DM, and overt diabetes is thought to be preceded by a long period of insulin resistance, during which blood glucose is maintained near normal levels by compensatory hyperinsulinemia (63). When cells are no longer able to compensate for insulin resistance by adequately increasing insulin production, impaired glucose tolerance (IGT) appears. This condition is characterized by an excessive blood glucose concentration in the postprandial phase, with fasting normal range. Persistence of imbalance between caloric intake and expenditure eventually leads to overt diabetes, characterized by high glycemia in any condition whether fasting or postprandial (63). 

Because evidence suggests that over nutrition, insulin resistance, IGT, diabetes, and CVD share in common the presence of an oxidative stress (65-67), in this review article, oxidative stress generation is proposed as the common persistent pathogenic factor mediating the appearance of insulin resistance as well as the passage from insulin resistance to overt diabetes, via IGT, while producing the increased cardiovascular risk condition typical of prediabetic and diabetic subjects by favoring atherosclerotic complications. This hypothesis may help us understand why diverse therapeutic interventions, which have in common the ability to reduce oxidative stress, can impede or delay the onset of diabetes and CVD.

***Production of ROS in Diabetes***

One unifying mechanism of injury in diabetes lies in the ability of both metabolic and vascular insults to increase cellular oxidative stress and impair the function of mitochondria (68, 69). Recent studies have supported this hypothesis, including *in vivo *and *in vitro *measurement of oxidative stress in sensory neurons as well as neuronal protection by antioxidants. * In vitro*, application of 10–20 mm glucose to dorsal root ganglia neurons leads to production of O2^_. ^and H2O2 that leads to lipid oxidation and neuronal death. This glucose-induced death is prevented by IGF-I, in part through decreased ROS production (70). Further evidence comes from feeding mice with a high-glucose diet. In this case, the mice experience hyperglycemia that leads to free radical production and oxidative stress (71). There is a close correlation between oxidative stress in diabetes and the development of complications. In type 1 diabetic patients, oxidative stress is evident within a few years of diagnosis before the onset of complications. As the disease progresses, antioxidant potential decreases, and plasma lipid peroxidation products increase depending upon the level of glycemic control (72). Type 2 diabetic patients have increased lipid peroxidation compared with age matched control subjects, as well as decreased plasma GSH and GSH-metabolizing enzymes and antioxidant potential, all of which relate directly to the rate of development of complications (73, 74). Similarly, oxidative stress is linked to preclinical features of disease, such as vascular endothelial activation that can lead to atherosclerosis (75). The lowered total antioxidant capacity may impair the protection against ROS and RNS causing O&NS and damage to fatty acids, proteins, DNA and mitochondria. The O&NS modified epitopes may mount an autoimmune response against these neoepitopes, which further may aggravate the initial inflammatory response These pathways in turn may induce depression and the (neuro) degenerative processes that accompany depression (figure 2) (76).

**Figure 2:**
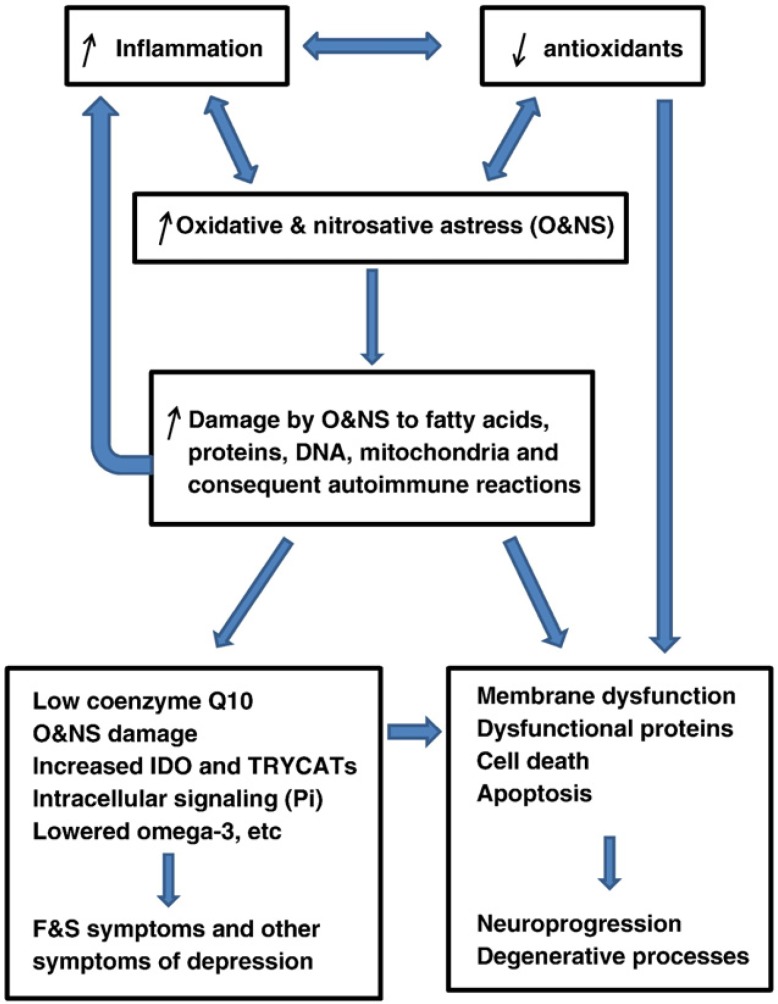
Oxidative and nitrosative stress (O&NS) (76).

***Biomarkers of Oxidative Stress***

Measuring biomarkers of oxidative stress is an essential step toward better a understanding of the pathogenesis and developing treatments for diabetic neuropathy. There are several approaches that may be adopted, including measurements of the depletion of antioxidant reserves, changes in the activities of antioxidant enzymes, free radical production, and presence of protein, lipid, and DNA free radical adducts. For the purposes of clinical assessment, measurements of end products of free radical attack may be the most reliable determination of the occurrence of oxidative stress because enzyme activities and cellular antioxidants are likely to display transient changes. Yet, the other measures also have utility depending on the nature of the study. The presence of oxidative stress in biological fluids can be simply assessed by examination of spontaneous visible luminescence. This phenomenon is the result of oxidized biomolecules with long half-life luminescent intermediates (77). 

Measures of spontaneous luminescence increased in the urine of patients with known oxidative stress such as hyperthyroid and muscular dystrophy patients or smokers compared with healthy controls (78). At present, this method is not routinely used in diabetes studies, because more specific end points are selected. These analyses can be performed not only on tissue but also on plasma, urine and saliva. Urine analysis can reveal nitrosylated proteins (79), lipid oxidation products such as 8-isoprostanes (80), and the DNA adduct 8-hydroxy-2-deoxyguanosine (8-OH-2dG) (81). These three indicators, along with other lipid adducts, *i.e*., malondialdehyde and 4-hydroxynonenyl and carbonyl derivatives of protein side chains, constitute the most common markers of oxidative stress in biological systems. Generally, measures of antioxidants or oxidized end products are more consistently performed in plasma than urine (1-12, 82). The excretion of 8-OH-2dG in urine may be misleading, because this parameter is more strongly influenced by the degree of oxygen consumption and activity of xenobioticmetabolizing enzymes (83). 

Blood cell 8-OH-2dG is increased in both type 1 and type 2 diabetic patients (84).

Carbonylated proteins and peptides are also inactivated by oxidative stress (85). Measurements of protein carbonyls are highly sensitive, and they can be detected in the plasma of both type 1 and type 2 diabetic patients even without complications (86, 87).

***Role of Oxidative Stress in Insulin Resistance***

The most important tissues involved in the pathogenesis of insulin resistance are muscle and adipose tissue. When caloric intake exceeds the energy expenditure, the substrate-induced increase in citric acid cycle activity generates an excess of mitochondrial NADH (mNADH) and reactive oxygen species (ROS) (88). To protect themselves against harmful effects of ROS, cells may reduce the formation of ROS and/or enhance ROS removal. Prevention of ROS formation is accomplished by preventing the build-up of mNADH by inhibiting insulin stimulated nutrient uptake and preventing the entrance of energetic substrates (pyruvate, fatty acids) into the mitochondria. Controversy exists as to whether free fatty acid (FFA) or glucose is the primary fuel source in the over nourished muscle and adipose tissue. In either case, an influx of substrates into the citric acid cycle generates mitochondrial acetyl-CoA and NADH (88). Acetyl-CoA, derived either from glucose through pyruvate or from beta-oxidation of FFA, combines with oxaloacetate to form citrate, which enters the citric acid cycle and is converted to isocitrate. NAD-dependent isocitrate dehydrogenase generates NADH. When excessive NADH cannot be dissipated by oxidative phosphorylation (or other mechanisms), the mitochondrial proton gradient increases and single electrons are transferred to oxygen, leading to the formation of free radicals, particularly superoxide anion (89, 90) .The generation of excessive NADH may be prevented in several ways, one of which is the inhibition of FFA oxidation (32). An increase in intracellular FFA, in turn, leads to reduced GLUT4 translocation to the plasma membrane, resulting in resistance to insulin stimulated glucose uptake in muscle and adipose tissue. In this setting, insulin resistance may be considered a compensatory mechanism that protects the cells against further insulin stimulated glucose and fatty acid uptake and therefore oxidative damage (91, 92). Many studies support this hypothesis: in vitro studies and in animal models, antioxidants have been shown to improve insulin sensitivity (90, 93).

***Role of Oxidative Stress in Dysfunction of Beta and Endothelial Cells***

It is a reasonable hypothesis that what happens in muscle and fat cells may also occur in other cells, particularly in beta-cells and endothelial cells. Moreover, these cell types may be particularly affected by overfeeding. These cells are notably not dependent on insulin for glucose uptake, which here is via facilitative diffusion instead of insulin-regulated glucose transporters. Therefore, if overfed, they cannot down-regulate the influx of nutrients by means of insulin resistance, and must allow intracellular concentrations to increase further. Many studies have suggested that beta-cell dysfunction results from prolonged exposure to high glucose, elevated FFA levels, or a combination of both (93). Beta-Cells are particularly sensitive to ROS because they are low in free-radical quenching (antioxidant) enzymes such as catalase, glutathione per- oxidase, and superoxide dismutase (94). Therefore, the ability of oxidative stress to damage mitochondria and markedly blunt insulin secretion is not surprising (95). 

Recent studies have suggested that beta-cell lipotoxicity is enhanced byconcurrent hyperglycemia and that oxidative stress may be the mediator (96, 97). The response-to-injury hypothesis of atherosclerosis states that the initial damage affects the arterial endothelium in terms of endothelial dysfunction (98). Notably, today’s evidence confirms that endothelial dysfunction, associated with oxidative stress, predicts cardiovascular disease (99, 100). 

Role of Oxidative Stress in Inflammation, Insulin Resistance, Diabetes, and CVD

Although the concept of atherosclerosis as an inflammatory disease is now well established, line of evidence suggests that chronic inflammation may be involved in the pathogenesis of insulin resistance and T2DM. This lead to the hypothesis that inflammatory changes may be considered a common pathogenic step in all of these conditions (10, 11, 101). 

The concept that oxidative stress is the common factor underlying insulin resistance, T2DM, and CVD, and may explain the presence of inflammation in all these conditions (2, 4, 9, 102, 103). It is well recognized that inflammation is one manifestation of oxidative stress, and the pathways that generate the mediators of inflammation, such as adhesion molecules and interleukins, are all induced by oxidative stress (104).

## Conclusion

In conclusion, a puzzle of many pieces of evidence suggests that free radical over generation may be considered the key in the generation of insulin resistance, diabetes, and cardiovascular disease.
